# Led Astray by Hemoglobin A1c: A Case of Misdiagnosis of Diabetes by Falsely Elevated Hemoglobin A1c

**DOI:** 10.1177/2324709616628549

**Published:** 2016-01-28

**Authors:** Jean Chen, Amy Diesburg-Stanwood, Geza Bodor, Neda Rasouli

**Affiliations:** 1 University of Colorado School of Medicine, Aurora, CO, USA; 2 VA Eastern Colorado Health Care Systems, Denver, CO, USA

**Keywords:** diabetes mellitus, hemoglobin A1c, hemoglobin Wayne, high-performance liquid chromatography

## Abstract

Hemoglobin A1c (A1c) is used frequently to diagnose and treat diabetes mellitus. Therefore, it is important be aware of factors that may interfere with the accuracy of A1c measurements. This is a case of a rare hemoglobin variant that falsely elevated a nondiabetic patient’s A1c level and led to a misdiagnosis of diabetes. A 67-year-old male presented to endocrine clinic for further management after he was diagnosed with diabetes based on an elevated A1c of 10.7%, which is approximately equivalent to an average blood glucose of 260 mg/dL. Multiple repeat A1c levels remained >10%, but his home fasting and random glucose monitoring ranged from 92 to 130 mg/dL. Hemoglobin electrophoresis and subsequent genetic analysis diagnosed the patient with hemoglobin Wayne, a rare hemoglobin variant. This variant falsely elevates A1c levels when A1c is measured using cation-exchange high-performance liquid chromatography. When the boronate affinity method was applied instead, the patient’s A1c level was actually 4.7%. Though hemoglobin Wayne is clinically silent, this patient was erroneously diagnosed with diabetes and started on an antiglycemic medication. Due to this misdiagnosis, the patient was at risk of escalation in his “diabetes management” and hypoglycemia. Therefore, it is important that providers are aware of factors that may result in hemoglobin A1c inaccuracy including hemoglobin variants.

## Introduction

Hemoglobin A1c (A1c) became commercially available in 1978, and the American Diabetes Association recommended its use in 1994 to assess the effectiveness of management on glycemic control by providing specific A1c goals.^1^ In 2010, the American Diabetes Association added A1c to clinical guidelines as a means for diagnosis of diabetes.^2^ Because A1c does not require fasting and can be drawn at any time of day, providers may feel that it is a more convenient tool for diagnosis of diabetes as compared to the fasting plasma glucose (FPG) test and the oral glucose tolerance test (OGTT).^3,4^

Currently, one could diagnose diabetes mellitus based on 2 consecutive measurements of A1c ≥6.5% with no formal recommendations to confirm the diagnosis with alternative testing, such as FPG or an OGTT.^5^ In this article, we present a case of falsely elevated A1c levels due to a rare hemoglobin variant, which led to the misdiagnosis of diabetes.

## Case Report

A 67-year-old Caucasian male with Hashimoto’s thyroiditis and spinal stenosis was referred to the endocrine clinic for the management of uncontrolled diabetes mellitus. His primary care provider had diagnosed him with type 2 diabetes based on an elevated A1c of 10.7%, which is equivalent to an average blood glucose of 260 mg/dL. Metformin 500 mg twice daily was started and titrated to 1000 mg twice daily after one week. At the initial endocrine visit, the patient complained of fatigue, weight loss, and intermittent abdominal pain.

**Figure 1.**
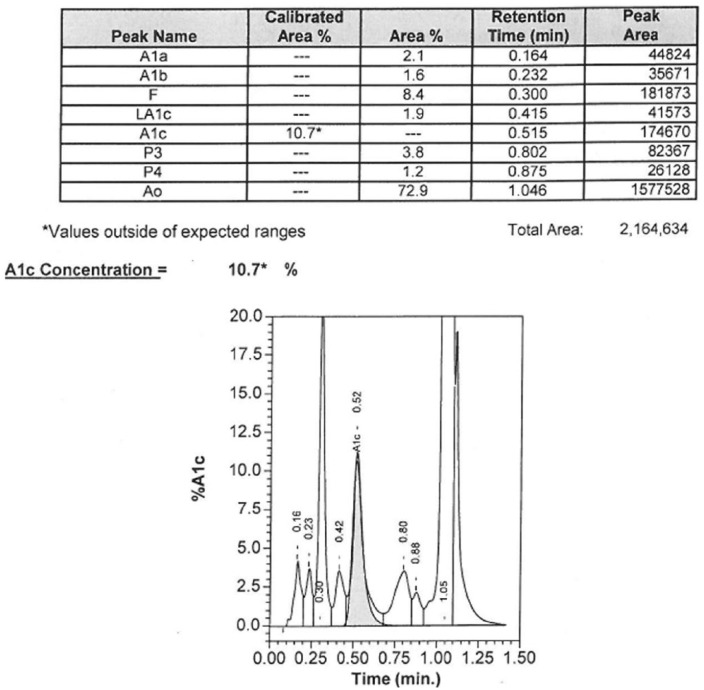
A1c measurement by cation-exchange high-performance liquid chromatography (BioRad Variant II HPLC analysis).

Despite already having a low body mass index of 18, lifestyle changes were implemented, including a very low carbohydrate diet. The patient had not been monitoring his blood glucose at home. While instructing the patient on how to use a glucometer, it was noted that he had a nonfasting capillary blood glucose of 100 mg/dL. In the absence of metabolic syndrome and given his previous high A1c with normal glucose level, decision was made to evaluate him for type 1 diabetes entering the honeymoon phase (transient β-cell remission). The patient had a C-peptide of 2.5 ng/mL with blood glucose of 102 mg/dL and negative glutamic acid decarboxylase antibodies (less than 1.0 IU/mL). These results did not support β-cell dysfunction or autoimmunity against the β-cells. Additionally, his fructosamine level was 223 µmol/L (reference range = 0-285 µmol/L), which did not reflect hyperglycemia and was consistent with his home glucose measurements of 92 to 130 mg/dL (fasting and pre/post meals). However, his A1c continued to be 10% to 11% using the BioRad Variant II high-performance liquid chromatography (HPLC) analysis (Figure 1); therefore, further investigation into the cause of this false elevation was done.

There was no history or laboratory result to support the more commonly known causes of falsely elevated A1c. These would be conditions that can decrease red blood cell (RBC) turnover, such as asplenia, B_12_ deficiency anemia, or folate deficiency anemia.^1^ He had no history to suspect the presence of a nonfunctioning spleen nor had he had a splenectomy. The patient also had normal B_12_ and folate levels. Though the mechanism has not been clearly identified, studies have shown an association between iron deficiency anemia and elevated A1c levels in nondiabetic patients.^6-8^ The patient did not suffer from anemia nor had iron studies consistent with iron deficiency. Severe hypertriglyceridemia^9^ and severe hyperbilirubinemia have also been previously reported to cause false elevation of A1c.^1^ However, this patient had a nonelevated fasting triglyceride and normal bilirubin level. Laboratory findings are summarized in Table 1.

**Table 1.** Lab Results.

HGB	14.6 (14.5-18.1 g/dL)
HCT	44.8 (42-54%)
MCV	89.7 (80-100 fL)
Vitamin B_12_	838 (220-600 pg/mL)
Folate	809 (499-1504 ng/mL)
Iron	54 (40-150 µg/dL)
TIBC	322 (280-500 µg/dL)
Ferritin	117.9 (40-400 ng/mL)
Triglycerides	108 (≤149 mg/dL)
Total bilirubin	0.5 (<1.2 mg/dL)
BUN	28 (8-22 mg/dL)
Creatinine	1.0 (0.6-1.3 mg/dL)

Abbreviations: HGB, hemoglobin; HCT, hematocrit; MCV, mean corpuscular volume; TIBC, total iron building capacity; BUN, blood urea nitrogen.

**Figure 2.**
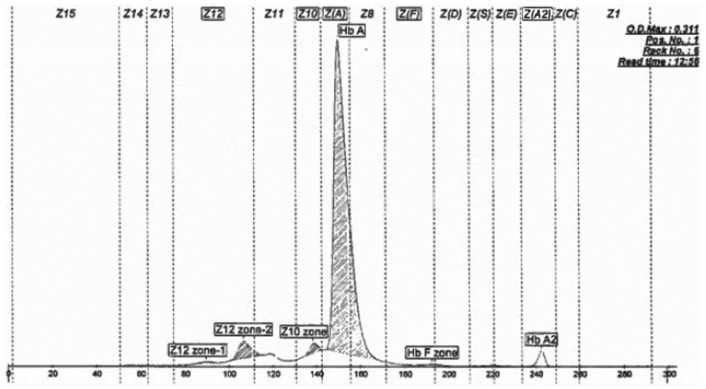
Hemoglobin electrophoresis. Hb A = 90.4%; Hb A2 = 2.3%; Hb F zone = 0.3%; Z12 zone-1 = 0.5%; Z12 zone-2 = 5.0%; Z10 zone = 1.5%. Spikes in Z10 zone, Z12 zone-1, and Z12 zone-2 are consistent findings in the presence of hemoglobin Wayne.

Review of patient’s A1c chromatography (Figure 1) revealed a hemoglobin fraction in the F-window (retention time of 0.3 minutes) and caused concern for hereditary persistence of hemoglobin F (HPHF). Hemoglobin electrophoresis was ordered to evaluate for hemoglobin variants, another known cause of falsely elevated A1c levels. Hemoglobin electrophoresis did reveal the presence of a hemoglobin variant but was not consistent with hemoglobin F or other commonly known variants, such as Hb S or C (Figure 2). Therefore, genetic sequencing was performed and identified a hemoglobin A mutation. This mutation consisted of a nucleic acid deletion (C.420delA), which resulted in an amino acid alteration of Lys139Asnfs and the extension of the affected α-globin molecule by 5 amino acids. This hemoglobin variant has been reported previously and referred to as hemoglobin Wayne.^10-12^

After revealing the presence of this hemoglobin variant, boronate affinity chromatography was used to measure A1c, which was found to be 4.7%. This value was more consistent with reported blood glucose levels and further supported the fact the patient indeed did not have diabetes.

## Discussion

It is known that certain hemoglobin variants can confound A1c measurements through various mechanisms. In our case, hemoglobin Wayne caused inaccuracy because of the interference with the assay method that was used. At our facility, A1c is measured by cation exchange HPLC using the BioRad Variant II analyzer. This method has been shown to be susceptible to inaccuracy in the presence of hemoglobin variants.^13,14^

With cation-exchange HPLC, proteins are separated by its specific net charge. These net charges determine the elution rate, also known as retention time, of each protein traveling through a glass column. Based on these elution rates, the concentration of a specific protein can be measured. Hemoglobin Wayne has an amino acid substitution that confers a similar charge to A1c. Therefore, hemoglobin Wayne and hemoglobin A1c have similar retention time, causing an overestimation of A1c concentration. When the hemoglobin electrophoresis showed the presence of an uncommon hemoglobin variant, boronate affinity chromatography, an alternative method to measure A1c, was used. The boronate affinity method incorporates an extra step of washing out nonglycated hemoglobin A1c prior to HPLC.^15^ It does not rely on net charges. Therefore, it is able to measure A1c accurately in the presence of most hemoglobin variants, especially those that have a net charge similar to A1c. Our patient’s A1c by boronate affinity chromatography was 4.7% and confirmed the absence of diabetes.

Others have also documented how interference by hemoglobin variants on A1c measurement can be method dependent. Schnedl et al^16^ evaluated the effect of various hemoglobin variants (Hb Graz, Hb Sherwood Forest, Hb O Padova, Hb D, and Hb S) by measuring A1c using ion-exchange HPLC, boronate affinity chromatography, and immunoagglutination. This study demonstrated that HPLC and immunoagglutination methods had more difficulty than the boronate affinity chromatography in terms of reporting an accurate A1c level. Nyenwe and Fischer^17^ described falsely elevated A1c when using an ion-exchange HPLC in the presence of a hemoglobin variant named hemoglobin N-Baltimore. However, Lorenzo-Medina et al^18^ reported little interference from hemoglobin N-Baltimore when using an immunoagglutination method to measure A1c.

Besides being effected by the presence of hemoglobin variants, there are other limitations in the use of A1c to identify undiagnosed diabetes. The concerns include the appropriate cutoff for the diagnosis of diabetes and variations in A1c levels among different ethnic groups.^19-21^ Also, just as for causes of falsely elevated A1c, common causes of falsely decreased A1c should be kept in mind. Inaccurately low A1c can be seen in conditions that increase RBC turnover, such as splenomegaly, acute and chronic blood loss and hemolytic anemia, or conditions that shorten RBC lifespan, such as end-stage renal disease.^1^

Concern should arise when blood glucose levels do not correlate with the A1c values. In those cases, there may be utility in using alternative laboratory tests, such as FBG, OGTT, or fructosamine to support or refute the diagnosis of diabetes. Though OGTT was not felt to be necessary in this case, it would have provided a definitive answer to if this patient truly had the degree of hyperglycemia that was reflected by the elevated A1c.

## Conclusion

Hemoglobin A1c can now be used to diagnose prediabetes and diabetes mellitus, reflect long-term blood glucose concentrations, and assess the risk of microvascular complications associated with diabetes. Therefore, A1c can contribute greatly to a provider’s decision to begin or aggressively intensify diabetes management. Thus, the purpose of this case was not only to present this rare and interesting hemoglobin variant but also serve as a reminder that A1c testing is susceptible to misinterpretation, especially in the presence of a hemoglobin variant or more common interfering factors. With the proper awareness, providers may avoid the mismanagement of both diabetic and nondiabetic patients.
